# The vitamin D grey areas in pediatric primary care. Very low serum 25-hydroxyvitamin D levels in asymptomatic children living in northeastern Italy

**DOI:** 10.1186/1687-9856-2012-7

**Published:** 2012-04-18

**Authors:** Stefano Mazzoleni, Daniela Toderini, Chiara Boscardin

**Affiliations:** 1 MD, Pediatra di Libera Scelta, ULSS 16 Regione Veneto, Padova, Italy; 2 MD, Endocrinologa, Medico di Medicina Generale, ULSS 16 Regione Veneto, Padova, Italy; 3 MD, Borsista Clinica Pediatrica, Università di Padova, Padova, Italy; 4 Correspondence address: Polistudio Pediatrico, via G. D'Annunzio 3/A, I-35028 Piove di Sacco (Padova), Italy

**Keywords:** 25-Hydroxyvitamin D, vitamin D, cholecalciferol, deficiency, insufficiency, supplementation

## Abstract

The principal questions about the vitamin D topic are far to be resolved: in which children 25-hydroxyvitamin D blood testing is appropriate and how much cholecalciferol should be given in the absence of the test? Analyzing vitamin D status in a group of children cared by a "family pediatrician" in northeastern Italy we noted a high incidence of deficiency in asymptomatic preschool children without risk factors. As routine vitamin D testing is not recommended in the average risk population the supplementation with cholecalciferol represents a "grey area" mostly in pediatric primary care.

## 

Dear Editor,

we read with interest the article by Bener and Hoffmann on the incidence of nutritional rickets in a sun rich country like Qatar [1], where decreased vitamin D was a major risk factor. Hypovitaminosis D is highly prevalent in children throughout the world [2,3] but it is still not clear what is the best practice in pediatric primary care settings. Michael Holick, a recognized expert on the topic, has stated that "there is no need to measure everybody's blood 25-hydroxyvitamin D" [25(OH)D] and that only patients with particular diseases should be screened for vitamin D insufficiency/deficiency [4]. Although the literature has shown that patients with deficiency are much less frequent than those with insufficiency, it is also remarkable that vitamin D deficiency is often subclinical and depending on local situations; for example it may be associated with overweight [5] or underweight [1]. To our knowledge there are only a few studies on children living in northeastern Italy [6-8]: they have been conducted retrospectively [6] or examining patients afferent to a Pediatric Department [7] or asthmatic [8]. On this basis an analysis of vitamin D status was prospectively conducted in children cared by a "family pediatrician" in a rural area near Padua (45° N latitude). In 65 patients the vitamin D test was included in exams ordered for different reasons (suspected anemia, fatigue, poor growth, etc.) between November 2010 and June 2011. Results were retrieved from 58 children (age range 1.1-15.3 years, median age 6.75 years). Serum 25(OH)D was dosed by chemiluminescence; the laboratory normal range was 75-250 nmol/l (30-99 ng/ml); insufficiency was defined as 25-74 nmol/l (10-29 ng/ml), deficiency as < 25 nmol/l (< 10 ng/ml).

Most of the children (77%) had low serum 25(OH)D levels: 38 of them (66% of all patients) had an insufficiency and 7 (12%) had a deficiency. Moreover, 29 children (50%) had 25(OH)D < 50 nmol/l (< 20 ng/ml) that is the cut-off recently suggested to diagnose vitamin D deficiency [5,9].

None among the 9 young teens (11-15 years) had a normal value of 25(OH)D and 6 of the 7 children with 25(OH)D < 25 nmol/l (< 10 ng/ml) were between ages 2 and 5 years; this deficiency was asymptomatic in 5/6 cases. Moreover, our children with 25(OH)D ≥ 75 nmol/l (≥ 30 ng/ml) and those with deficiency didn't differ for exposure to sunlight, food consumption, gender, ethnicity or BMI. Vitamin D status was also irrespective of other blood test results (see Table 1).

**Table 1:** Vitamin D status of cases based on laboratory reference ranges (plain text columns) and on recent literature [5,9] (bold columns)

	n	Sufficiency	Insufficiency	Deficiency	Insufficiency	Deficiency
		75-250 nmol/l	25-74 nmol/l	< 25 nmol/l	**50-74 nmol/l**	**< 50 nmol/l**

		30-99 ng/ml	10-29 ng/ml	< 10 ng/ml	**20-29 ng/ml**	**< 20 ng/ml**

1-5 years	27	8 (30%)	13	6	**7**	**12**

6-10 years	22	5 (23%)	17	0	**5**	**12**

11-15 years	9	0	8	1	**4**	**5**

Total	58	13 (23%)	38	7	**16**	**29**

Gender (M/F)	40/18	9/4	31/14			
		
Overweight	9	3	6			
		
Underweight	7	0	7			
		
Immigrant	11	3	8			
		
Normal exams	23	2	21			

Holick has suggested that "it would be much more cost-effective to implement a vitamin D supplementation program for all children and adults" [4] but the question now is how much vitamin D should be given. If 400 IU cholecalciferol per day may be sufficient in the first year of life [10], much more is needed in older children, assuming that most of them have less or much less than the minimum desirable [11]. Moreover, recommended doses of 600 IU per day [12] probably offer no advantage to children with 25(OH)D < 25 nmol/l (< 10 ng/ml) [13]. Although authoritative guidelines state that routine vitamin D testing is not warranted in the average risk population, the Holick's D-lemma [4] is far from being resolved.

## Abbreviations

25(OH)D: 25-hydroxyvitamin D.

## Competing interests

The authors declare that they have no competing interests.

## Authors' contributions

MS performed the statistical analysis and interpretation of data and drafted the manuscript. TD participated in the design of the study, in interpretation of data and final approval of the manuscript. BC participated in the collection of data, in the statistical analysis and in the drafting of manuscript. All authors read and approved the final manuscript.
